# MACT: A Manageable Minimization Allocation System

**DOI:** 10.1155/2014/645064

**Published:** 2014-02-23

**Authors:** Yan Cui, Huaien Bu, Hongwu Wang, Shizhong Liao

**Affiliations:** ^1^School of Computer Science and Technology, Tianjin University, 92 Weijin Road, Nankai District, Tianjin 300072, China; ^2^Department of Common Required Courses, Tianjin University of Traditional Chinese Medicine, 312 Anshanxi Road, Nankai District, Tianjin 300193, China; ^3^College of Traditional Chinese Medicine, Tianjin University of Traditional Chinese Medicine, 312 Anshanxi Road, Nankai District, Tianjin 300193, China

## Abstract

*Background*. Minimization is a case allocation method for randomized controlled trials (RCT). Evidence suggests that the minimization method achieves balanced groups with respect to numbers and participant characteristics, and can incorporate more prognostic factors compared to other randomization methods. Although several automatic allocation systems exist (e.g., randoWeb, and MagMin), the minimization method is still difficult to implement, and RCTs seldom employ minimization. Therefore, we developed the minimization allocation controlled trials (MACT) system, a generic manageable minimization allocation system. *System Outline*. The MACT system implements minimization allocation by Web and email. It has a unified interface that manages trials, participants, and allocation. It simultaneously supports multitrials, multicenters, multigrouping, multiple prognostic factors, and multilevels. *Methods*. Unlike previous systems, MACT utilizes an optimized database that greatly improves manageability. *Simulations and Results*. MACT was assessed in a series of experiments and evaluations. Relative to simple randomization, minimization produces better balance among groups and similar unpredictability. *Applications*. MACT has been employed in two RCTs that lasted three years. During this period, MACT steadily and simultaneously satisfied the requirements of the trial. *Conclusions*. MACT is a manageable, easy-to-use case allocation system. Its outstanding features are attracting more RCTs to use the minimization allocation method.

## 1. Background

The blind randomized control trial (RCT) is commonly accepted as the gold standard research method for evaluating medical innovations [1]. When correctly performed, the RCT ensures that the same sort of participants receive intervention and control, thus eliminating selection and confounding biases [2]. When the study size is large enough, unbiased allocation of participants into either treatment groups or control groups maintains the balance of numbers and prognostic factors of participants among groups. This is essential to establish the internal validity of an RCT and ensure that the results are objective and scientific. Unbiased allocation allows RCTs to evaluate the effectiveness of medical innovations, such as a new surgical operation or a drug, in a sample population. When the number of participants is small, unbiased allocation is not appropriate, and alternative allocation methods are required.

Methods for allocation of trial participants can be divided into two categories: randomized allocation that includes simple, stratified, and blocked randomizations and dynamic allocation that includes biased coin, urn designs, and minimization techniques. Although permuted block methods are increasingly popular, simple randomization is more widely used than dynamic methods. In fact, a 2001 review indicated that only 4% of 150 RCTs employed minimization methods [3].

The simple randomization method allocates participants according to a pregenerated random number table, random number generator, or methods similar to a coin toss. This represents a completely unpredictable approach. However, simple randomization may result in an imbalance of prognostic factors among groups when trial sizes are small [4].

Minimization, which was first proposed by Taves in 1974 [5], employs a deterministic algorithm to allocate participants into groups. This deterministic algorithm minimizes differences among groups with respect to prognostic factors associated with the entire trial. Literature indicates that minimization method achieves good group balance [4]. However, the nonrandom nature of the method may introduce selection bias, as it may be possible to predict which group the next subject will be enrolled in, provided the factor levels of the new subject are known [6]. To enhance the unpredictability of the minimization method, a random element has been introduced. A random element is a probability (*p*) value ranging from 0.5 to 1 [7]. After the initial minimization allocation, a participant is assigned to a group based on the probabilities for *p*. Other methods may be used to reduce predictability, such as excluding exact details of the algorithm in the protocol, collecting data that is not used as a prognostic factor at the time of randomization, or introducing site as a factor in a multisite trial.

Evidence suggests that minimization allocation outperforms simple randomization, resulting in less chance of an imbalance of prognostic factors and treatment factors among groups. As minimization is increasingly used to allocate participants in RCTs, these observations strengthen the credibility of trial results. However, the allocation of future patients to a trial is less predictable in simple randomization allocation compared to minimization [8]. Previously, a simulation study showed that the performance of simple randomization was similar to minimization when random element was applied [9].

Currently, there are few random allocation systems available. Kenjo et al. created an easily customized and multi-institutional minimization allocation system [10]. This system is based on the Practical Extraction and Report Language (PERL) for writing common gateway interface (CGI) script. This system balances prognostic factors among groups. However, details describing the system are limited. Cai et al. developed a generic minimization random allocation and blinding system coded with Microsoft Visual Basic and Active Server Pages (ASP) programming languages [11, 12]. System details, usage, and a portion of the code are available, but the design of the database is complex, resulting in difficulties associated with programming and maintenance. Furthermore, trial management is confusing, and system administration is difficult. Morice developed randoWeb, an online randomization tool for clinical trials [13]. This system provides simple, stratified, and dynamic randomization methods, but system management details are limited.

We sought to facilitate case allocation and RCT management through the development of a minimization allocation controlled trial (MACT) system. In this system, multicenter, multigrouping, multiple prognostic factors, and multilevel RCTs with simple randomization or minimization can be achieved. Benefits of this system include the use of an optimized common relational database based on just 4 tables, rather than an entity-attribute-value (EAV) model [14]. The common relational database provides a unified programming and management interface that makes MACT easy to generalize. The MACT codes are concise and easy to understand, which makes the final system operation, maintenance, and management very convenient. In theory, the maximum number of prognostic factors in MACT is limited by the capability of the hardware. In reality, the maximum number of prognostic factors is limited by the study design, as increasing numbers of prognostic factors make it difficult to control balance and predictability. In practice, the actual number of prognostic factors is much smaller than MACT's capability.

## 2. System Outline

### 2.1. System Architecture

#### 2.1.1. Software and Hardware

MACT is designed to run on a Windows 2000 platform and microcomputers that are able to support the MACT system. In practice, our platform is a DELL SC440 Server with 1 GB RAM, 160 GB hard disk, and a Pentium dual-core CPU E2180 @ 2.0 GHz.

User and trial management employs the internal IIS server of Windows 2000. E-mail requires a SMTP server and the DBMS is SQL Server 7.0.

Users can access MACT through any internet browser application; tested browsers include Internet Explorer and Chrome.

#### 2.1.2. System Architecture Diagram

**Figure 1:**
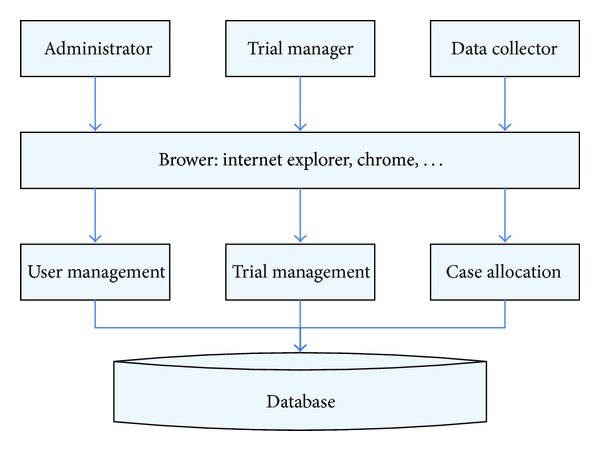
System architecture.

A schematic of MACT system architecture is shown in Figure 1. All users, including administrators, trial managers, and data collectors, utilize browsers. They access certain modules to complete their respective user management, trial management, and participant allocation tasks (Figure 1).

### 2.2. User Management

MACT has three tiers of management: administrator, trial manager, and data collector. In addition, there are two other classes of users: registered users and banned users. Registered and banned users are unable to complete any operations in MACT; however, registered users can be changed into data collectors or trial managers, but banned users cannot. Preassigned permissions are encoded as shown in Table 1.

The administrator has the most privileges. The administrator has the ability to access all modules and manages users through user management modules. Administration tasks include assigning data collectors to trials; adjusting permissions (e.g., changing users permissions to data collector or trial manager); and banning offending users. New users can register by telephone, short message service (SMS; text), or email and administrators can adjust permissions.

### 2.3. Trial Management

Trial managers design studies, register participants, and assign prognostic factors, numbers of groups, and *p*. Initialization of new trials involves

- entering the name of the trial in the data collection interface,
- setting the *p* to enhance the unpredictability of the minimization method,
- adding the important prognostic factors and identifying the associated levels in each factor to determine inclusion and exclusion criteria,
- assigning group names to treatment regimens (e.g., A, B, C, etc.).

Trial managers can view the total number of participants included in a trial, the number of participants recruited in each subcenter, and the number of participants in each group. Trial managers can communicate with data collectors via email.

### 2.4. Participant Allocation

Data collectors at each trial subcenter are responsible for recruiting participants. Data collectors enter participant information into MACT, and the system automatically allocates the participant to a group. Details of the allocation process are communicated to the data collectors and the trial manger via email. Data collectors are blinded to the specifics of the treatment regimens.

Patient privacy is protected by hardware and software firewalls. Access to patient data is restricted through appropriate MACT management procedures.

## 3. Methods

### 3.1. Database Design

Unlike previous systems, MACT employs a traditional relational database instead of an EAV database. EAV databases are often used in instances where the amounts of attributes, properties, or parameters that can be used to define an entity are potentially limitless, but sparse. EAV databases are especially applicable to RCTs, where they take into account different prognostic factors, levels, and allocation bias. Such differences result in heterogeneity. This heterogeneity is intensified if several RCTs exist within one system. The EAV design transforms the heterogeneity into one table that has three columns: entity, attribute, and value. Entity is the patient event and includes the patient ID; attribute or parameter is a foreign key into a table of attribute definitions; attribute definitions include an attribute ID, attribute name, description, data type, units of measurement, and input validation; value is the value of the attribute. The EAV database model has many advantages. First, it stores heterogeneous data through a unified logical structure that provides a convenient interface for programming. Second, it has a simple structure that is easily adaptable to accommodate multitrials. When new data arrives, it is appended directly to existing data without changing the structure of the table. Third, it efficiently utilizes storage space. However, the EAV model has two insurmountable shortcomings. First, the program has no ability to manipulate all the attributes within a whole row using one operation. Accessing a whole row in the EAV model requires many operations and multiple table joins. The process of querying an *n*-column row requires *n* SELECTs and *n* − 1 JOIN statements. In contrast, SQL data definition and query language used in most relational databases uses one SELECT statement. Second, EAV tables save storage space when the original table is sparse, but, when the occupancy rate is greater than 1/3, the EAV tables occupy a significantly higher amount of storage due to data redundancy.

**Figure 2:**
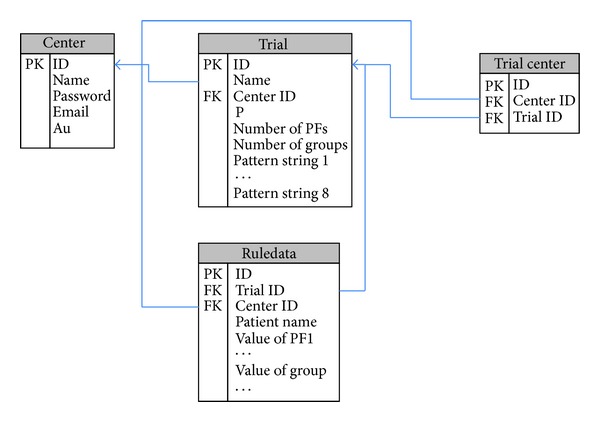
Database structure.

In order to improve the EAV model, MACT created a common relational database with a rule column (RTWR) to store trial data. MACT's database is illustrated in Figure 2.

The following is an explanation of the database and its various components.

*CENTER*. Stores information of all users (administrator, trial managers, data collectors, registered users, and banned users), including username, password, email addresses, and permission levels. Operations include registering users, changing permissions, and removing users. The latter two operations can only be performed by an administrator.

*TRIAL*. Stores information on all trials, including trial manager's ID, allocation bias, number of prognostic factors, the pattern string of every prognostic factor, and the number of groups. The pattern string contains the name, cutoff value, and weight of prognostic factors. The rule column in TRIAL contains the pattern string. The rule column in TRIAL and RULEDATA constitute the RTWR structure. This structure enables MACT to store heterogeneous data in RULEDATA.

*RULEDATA*. It stores all trial data, including trial ID, manager ID, participant names, and all values of prognostic factors. Data transformation uses a pattern string as follows.

- Information about prognostic factors can be acquired from TRIAL through trial ID.
- A preprogrammed conversion module acquires the name, cutoff values, and weight of prognostic factors from TRIAL and shows them on screen.

**Figure 3:**
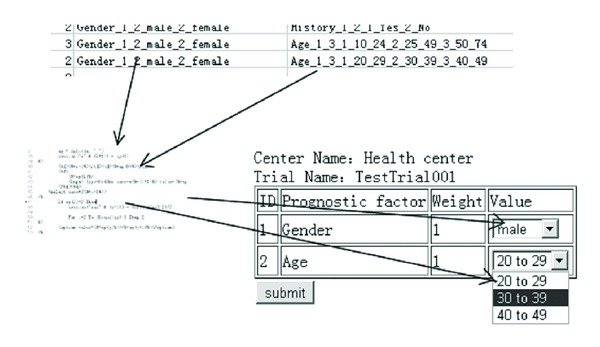
Rule translator.

For example, in Figure 3, the pattern string Age_1_3_1_20_29_2_30_39_3_40_49 is transformed: the name of the prognostic factor is Age; its weight is 1; it is divided into several levels, 20 to 29, 30 to 39, and 40 to 49. When data is needed, all of the information can be retrieved through the conversion module by one operation.

The common relational database has the ability to store heterogeneous data that can be retrieved by a single operation. However, the maximum number of prognostic factors is limited in this database, as the number of columns is unalterable; the maximum number of prognostic factors of all trials is equivalent to the number of columns in RULEDATA and storage space is wasted. As trials have different numbers of prognostic factors, there are empty columns in RULEDATA (Figure 3).

### 3.2. Minimization Allocation

The minimization method involves

- allocation of the first participant into an arbitrary group with the probability 1/number of groups,
- allocation of subsequent participants into group with probability *p*, such that arg min⁡_*h*_ *G*_*h*_ = ∑_*i*=1_^*M*^ *w*_*i*_ *D*_*ij*_, where *D*_*ij*_ is defined as *D*_*ij*_ = Var⁡(*x*_1*ij*_, *x*_2*ij*_,…, *x*_*kij*_), where *k* is the number of groups, *i* is the number of prognostic factors, *j* is the number of levels, *w* is the weight of prognostic factors, *p* is the allocation bias, and *x* is the number of case

Because minimization allocation and *p* allocation are two independent procedures, the minimization allocation transforms into simple random allocation when *p* = 0.5. In this situation, MACT performs simple randomization.

## 4. Simulations and Results

Single and multitrial simulations were performed to ensure that MACT would achieve the desired results. The single-trial simulation included 300 participants where *i* = 2, *j*_max⁡_ = 2, *k* = 2, *p* = 0.8, where *j*_max⁡_ represents the maximum value of prognostic factors. In the multitrial simulation, 3 RCTs were run simultaneously (Table 2). Balance of prognostic factors between intervention groups and the unpredictability of the minimization and simple randomization allocation methods were compared. Unpredictability was evaluated using Support Vector Machine (SVM) [15], a technique for bioinformatics classification [16–18]. In this task, SVM considered the allocations of the first 50 participants to predict allocation of the 51st participant. Subsequently, the 51st participant was added into the simulation and the above process was repeated until there were no new participants. The total numbers of correctly and incorrectly predicted results were recorded. The unpredictability is evaluated by the standard deviation of the number of correct and incorrect predicted allocation results. When the standard deviation is 0, the allocation is perfectly unpredictable.

### 4.1. Single Trial Simulation

Table 3 shows the balance of prognostic factors (PF) between intervention groups achieved by minimization and simple randomization in the single trial simulation. The values are standard deviations of participants among groups according to levels of prognostic factors. Smaller values indicate a smaller difference between groups; bold and italicized values indicate better performance (Tables 3–6). When 100 participants were allocated, minimization outperformed simple randomization in most cases. When 200 participants were allocated, minimization was not inferior to simple randomization in all cases and outperformed simple randomization in most cases. When 300 participants were allocated, the performance of minimization and simple randomization was similar, although minimization tended to outperform simple randomization.

We demonstrated the predictability of minimization and simple randomization in a single trial simulation in Table 4. The total numbers of correctly and incorrectly predicted results were also recorded (Table 4). Note that more incorrect predictions do not result in higher unpredictability because smart forecasters can use the opposite result of predictive algorithms. For this reason, we took the standard deviation from the number of correct and incorrect predictions as the measurement of unpredictability. When the first 100 participants were allocated, the unpredictability of minimization and simple randomization was the same in our simulation as indicated by the standard deviations. As the number of participants increased to 200 and 300, the unpredictability of minimization was greater than simple randomization.

### 4.2. Multitrial Simulations

The multitrial simulations confirmed that as the number of participants allocated increased, minimization achieved a better balance of prognostic factors among groups, while simple randomization had better unpredictability (Tables 5 and 6). Only the result of the largest trial, which is number 3 in Table 2, is recorded in Tables 5 and 6, because all the results are similar to the single trial.

## 5. Applications

MACT was initially employed for RCT 2006BAI08B02-01, which had four prognostic factors, including gender (2 levels), age (4 levels), primary disease (4 levels), and cardiac function (3 levels). In total, 340 participants were allocated into two groups and the allocation bias was 0.8. The MACT allocated participants are shown in Table 7. MACT achieved an ideal balance of prognostic factors between the two groups and 126 correct and 164 incorrect predictions.

Subsequently, MACT was employed for RCT 2008BAI53B04, which had three prognostic factors including gender (2 levels), age (7 levels), and medical score (10 levels). In total, 370 cases were allocated into two groups and the allocation bias was 0.8. There were 154 correct and 166 incorrect predictions (Table 8).

In practical applications, MACT achieved a good balance of prognostic factors among groups. This greatly improved the internal validity of the RCTs and yielded more robust conclusions.

## 6. Conclusions

MACT is an easy-to-manage allocation system. Currently, 11 hospitals in northern China are registered as subcenters. The trial managers and data collectors in these subcenters became familiar with the system within one hour's training. Trials are managed and participants are allocated without further programming. MACT has excellent stability. So far, MACT has run continuously for three years. With the exception of regular hardware maintenance, the system has never failed. As an easy-to-expand, easy-to-manage, and stable system, MACT facilitates the use of the minimization method in the practice of clinical trials.

## Figures and Tables

**Table 1:** User permissions coding.

Coding	Permissions
128	Administrator
64	Trial manager
32	Data collector
16	Reserved
8	Reserved
4	Reserved
2	Banned user
1	Registered user

**Table 2:** The setup of multitrial simulations.

Number	Number of prognostic factors	Number of maximum levels	Number of groups	Allocation bias
1	2	3	2	0.9
2	3	4	3	0.8
3	6	5	3	0.7

**Table 3:** The standard deviation of cases and prognostic factors among groups.

Number of cases	100		200		300	
Method	Minimization	Simple	Minimization	Simple	Minimization	Simple
Number of cases	***1.41***	7.07	***1.41***	7.07	***0.00***	1.41
PF1						
Level 1	***0 .705***	4.95	**0.705**	2.12	***0.00***	1.41
Level 2	2.12	2.12	***0.705***	4.95	***0.00***	2.83
PF2						
Level 1	2.12	2.12	***2.12***	9.19	4.95	4.95
Level 2	4.95	***0.705***	***4.95***	6.36	8.49	8.49
Level 3	***4.24***	5.56	4.24	4.24	***3.54***	4.95

**Table 4:** The unpredictability of a single trial.

Cases	100		200		300	
Method	Minimization	Simple	Minimization	Simple	Minimization	Simple
Correct	29	21	77	75	131	120
Incorrect	21	29	73	75	119	130
Correct%	42.0	58.0	51.3	50.0	52.4	48.0
SD	5.66	5.66	2.83	***0.00***	8.49	***7.07***

**Table 5:** The standard deviation of cases and prognostic factors among groups.

Number of Cases	300		400		500	
Method	Minimization	Simple	Minimization	Simple	Minimization	Simple
Number of cases	***0.816***	1.41	***0.816***	4.97	***0.816***	1.41
PF1						
Level 1	***0.816***	2.16	***0.577***	3.70	***0.500***	4.92
Level 2	***0.00***	1.83	***0.577***	2.65	***0.957***	3.59
Level 3	***0.500***	3.20	***0.500***	3.30	***1.41***	4.97
Level 4	***1.15***	2.58	***0.577***	2.38	***0.577***	2.65
Level 5	***0.957***	1.71	***0.500***	3.77	***0.00***	5.48
PF2						
Level 1	***0.957***	4.27	***0.816***	3.65	***0.577***	3.70
Level 2	***0.816***	3.92	***0.500***	6.99	***0.957***	11.9
Level 3	***0.957***	4.86	***0.500***	4.99	***0.500***	8.62
PF3						
Level 1	***0.816***	5.23	***0.00***	2.94	***0.500***	3.59
Level 2	***0.816***	6.32	***0.816***	6.38	***0.500***	9.57
PF4						
Level 1	***0.500***	2.99	***0.957***	4.50	***0.577***	3.70
Level 2	***0.500***	4.03	***0.957***	4.19	***0.816***	5.42
Level 3	***0.500***	2.87	***0.957***	2.22	***0.577***	4.43
Level 4	***0.957***	4.65	***0.00***	5.48	***0.577***	4.43
Level 5	***0.816***	2.94	***0.500***	2.63	***0.577***	2.52
PF5						
Level 1	***0.500***	5.50	***0.577***	7.54	***0.500***	6.80
Level 2	***0.957***	5.12	***1.00***	5.26	***0.816***	8.29
Level 3	***0.577***	2.38	***0.500***	3.70	***0.500***	5.85
Level 4	***0.816***	4.16	***0.710***	4.79	***0.816***	4.00
PF6						
Level 1	***0.577***	5.07	***2.06***	6.18	***4.11***	8.62
Level 2	***1.26***	2.36	***1.91***	4.43	***1.71***	5.32
Level 3	***1.71***	2.50	***2.50***	5.74	***4.12***	6.95

**Table 6:** The unpredictability of multitrials.

Cases	300		400		500	
Method	Minimization	Simple	Minimization	Simple	Minimization	Simple
Correct	42	63	67	89	86	113
Incorrect	208	187	283	261	364	337
Correct %	16.8	25.2	19.1	25.4	19.1	25.1
SD	117	***87.7***	153	***122***	197	***158***

**Table 7:** The allocation results of project 2006BAI08B02-01.

PF	Level	Group 1	Group 2
1	1	91	94
	2	78	77

2	1	10	9
	2	25	27
	3	45	46
	4	89	89

3	1	137	136
	2	7	9
	3	7	7
	4	18	19

4	1	21	20
	2	96	97
	3	52	54

Total		169	171

**Table 8:** The allocation results of project 2008BAI53B04.

PF	Level	Group 1	Group 2
1	1	121	122
	2	64	63

2	1	10	10
	2	48	46
	3	34	37
	4	33	34
	5	37	39
	6	18	14
	7	5	5

3	1	0	0
	2	3	3
	3	17	17
	4	78	78
	5	36	36
	6	23	24
	7	12	12
	8	8	7
	9	1	2
	10	7	6

Total		185	185
